# The Australian Paradox: A Substantial Decline in Sugars Intake over the Same Timeframe that Overweight and Obesity Have Increased

**DOI:** 10.3390/nu3040491

**Published:** 2011-04-20

**Authors:** Alan W. Barclay, Jennie Brand-Miller

**Affiliations:** 1 Australian Diabetes Council, 26 Arundel Street, Glebe, NSW 2037, Australia; Email: awbarclay@optusnet.com.au; 2 School of Molecular Bioscience and Boden Institute of Obesity, Nutrition and Exercise, University of Sydney, NSW 2006, Australia

**Keywords:** sugars, sucrose, obesity, epidemiology, Australia

## Abstract

Ecological research from the USA has demonstrated a positive relationship between sugars consumption and prevalence of obesity; however, the relationship in other nations is not well described. The aim of this study was to analyze the trends in obesity and sugar consumption in Australia over the past 30 years and to compare and contrast obesity trends and sugar consumption patterns in Australia with the UK and USA. Data on consumption of sugar in Australia, the UK and USA were obtained from the Food and Agriculture Organization for the years 1980-2003. The prevalence of obesity has increased 3 fold in Australians since 1980. In Australia, the UK and USA, per capita consumption of refined sucrose decreased by 23%, 10% and 20% respectively from 1980 to 2003. When all sources of nutritive sweeteners, including high fructose corn syrups, were considered, per capita consumption decreased in Australia (−16%) and the UK (−5%), but increased in the USA (+23%). In Australia, there was a reduction in sales of nutritively sweetened beverages by 64 million liters from 2002 to 2006 and a reduction in percentage of children consuming sugar-sweetened beverages between 1995 and 2007. The findings confirm an “Australian Paradox”-a substantial decline in refined sugars intake over the same timeframe that obesity has increased. The implication is that efforts to reduce sugar intake may reduce consumption but may not reduce the prevalence of obesity.

## 1. Introduction

The prevalence of overweight/obesity continues to rise around the globe, in both developed and developing nations. The World Health Organization estimates that there are currently more than 700 million overweight adults, and at least 300 million obese adults [1]. The health consequences of overweight/obesity are well documented, and include increased risk of cardiovascular disease, cancer (endometrial, breast, and colon), type 2 diabetes, respiratory problems and osteoarthritis [2]. The economic costs of overweight/obesity are as equally grave as the health consequences, but not as well described. However, in several developed countries, obesity has been estimated to account for 2-7% of the total health care costs [3].

The most recent population health surveys in Australia determined that in 2007-2008, 62% of Australia’s 15 million adults [4] and 23% of Australia’s 5 million children [5] were either overweight or obese. The direct financial cost of obesity was estimated to be AUD$8.283 billion in Australia in 2008. In addition to this, the cost of lost wellbeing due to obesity was valued at AUD$49.9 billion, bringing the total cost of obesity in Australia in 2008 to AUD$58.2 billion [6].

While the cause of this pandemic of overweight/obesity is complex, multi-factorial, and likely to vary from region-to-region, researchers continue to look for common environmental factors to help explain the phenomenon. Increasing consumption of sugars [7], and in particular sugar-sweetened beverages [8], has been identified as a plausible etiological factor in the United States. Little is known about the relationship between sugars consumption and obesity in other nations, however. 

The aim of this study was to examine in detail trends in obesity in Australia, and to analyze concurrent trends in sugars and sugar-sweetened beverage consumption, and to compare these to those in the United Kingdom (UK) and the United States of America (USA).

## 2. Methods

### 2.1. Literature Search

A systematic literature review was undertaken to obtain sources of Australian sugar intake data. Key words used in the search included: sugars, sucrose, dietary carbohydrate, consumption, intake, sugar-sweetened beverages, sweeteners, refined sugar, obesity, adiposity, body weight, body mass index (BMI) with Australia. The term “blood glucose” was excluded in the search strategy. The databases searched were MEDLINE, Cinahl, Embase and the Cochrane library. Full papers were retrieved if they included a healthy population as the sub-group and were relevant in the Australian context and published within the last 30 years (since 1980). 

In addition to the peer-reviewed literature, publications and data issued by government, academia and industry were also explored. The websites of the World Health Organization (WHO) [9], Food and Agriculture Organization (FAO) of the United Nations [9], Australian Bureau of Statistics [10], Australian Food and Grocery Council [11], Australian Retailers Association [12], Commonwealth Scientific and Industrial Research Organization (CSIRO) [13] and the Australian Government [14] were searched for relevant information. The Australian food industry including the Australian Beverage Council (Ltd.) [15], CSR (Ltd.) [16], Coles [17] and Woolworths [18] supermarkets were also contacted for relevant data. 

For this analysis, water based beverages were categorized as nutritively sweetened or non-nutritively sweetened. Nutritively sweetened beverages included all sugar-sweetened (cane or fruit sugar) beverages such as soft drinks, sports drinks, iced tea drinks and flavored waters, but Non-nutritively sweetened beverages were defined as plain still/mineral waters or beverages sweetened with non-nutritive sweeteners. 

### 2.2. Prevalence of Obesity

Obesity statistics describing the percentage of obesity in the study population using BMI ≥ 30 kg/m2 in adults and a BMI greater than or equal to the 95th percentile, using age and sex dependent reference values in children, were obtained. 

Annual trends in obesity prevalence were obtained for Australia, the UK and the USA from a variety of sources. In Australia, data were sourced from the Australian Institute of Health and Welfare Risk Factor Data [18] store which was based on an analysis of the 1980, 1983 and 1989 Risk Factor Prevalence Surveys [19]; the 1995 National Nutrition Survey [20]; the 1999-2000 Australian Diabetes, Obesity and Lifestyle study [21]; the Australian Bureau of Statistics National Health Survey’s of 2004-2005 [22] and 2007-2008 [4] and the 2007 Australian National Children’s Nutrition and Physical Activity Survey [5]. In the UK, data were obtained from the Health Survey for England 2007 Latest Trends [23] and for the USA, from the National Health and Nutrition Examination Survey data (NHANES) [24]. 

### 2.3. Sugars Consumption

Data on annual apparent consumption of sugar were obtained for Australia, the UK and the USA from the FAO [25]. Average population sugar, sugary foods and beverages intake estimates were obtained from the 2007 Australian National Children’s Nutrition and Physical Activity Survey [5,26] and food and nutrient intakes from the 1983, 1985 and 1995 National Nutrition Surveys [19]. Australian Bureau of Statistics population estimates [27] were used to obtain data for total per capita beverage sales. 

Dr. Gina Levy [28] provided supplementary water-based beverage volume sales data for the years 2005 and 2006. The Australian Beverage Council Ltd. [15] representing major water-based beverage companies such as Coca-Cola Amatil Australia, Pepsico Australia, Cadbury Schweppes Australia and Unilever Australasia, provided volume sales data for the 10 year period from 1994 to 2004. This sales data was formerly from AC Nielsen Scan Track Data [29]. 

## 3. Results

### 3.1. Obesity Prevalence

**Figure 1:**
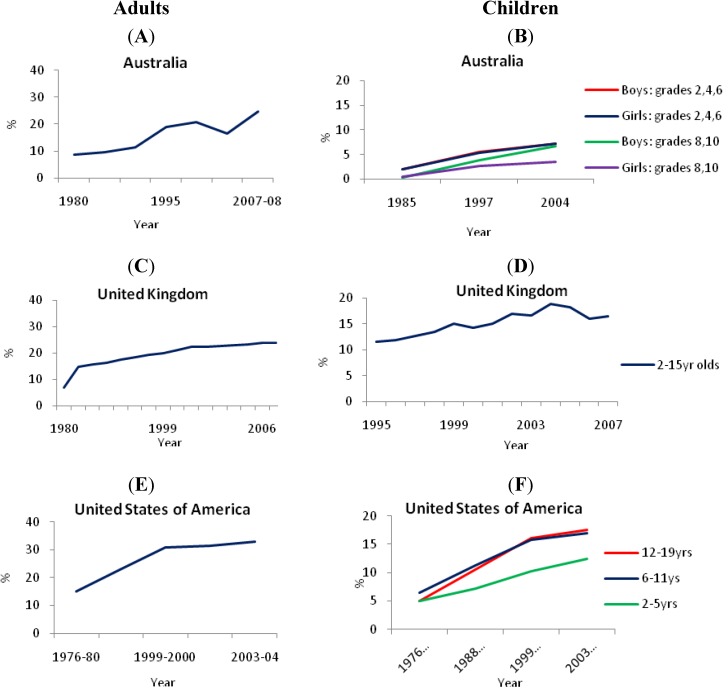
Prevalence of obesity (%) in (**A**) Australian adults, (**B**) Australian children, (**C**) adults in the United Kingdom, (**D**) children in the United Kingdom and (**E**) American adults (**F**) and children [18,23,24].

Obesity rates increased in Australia [18], the UK [23] and the USA [24], for adults, adolescents and children (Figure 1). 

### 3.2. Apparent Consumption of Sugar

**Figure 2:**
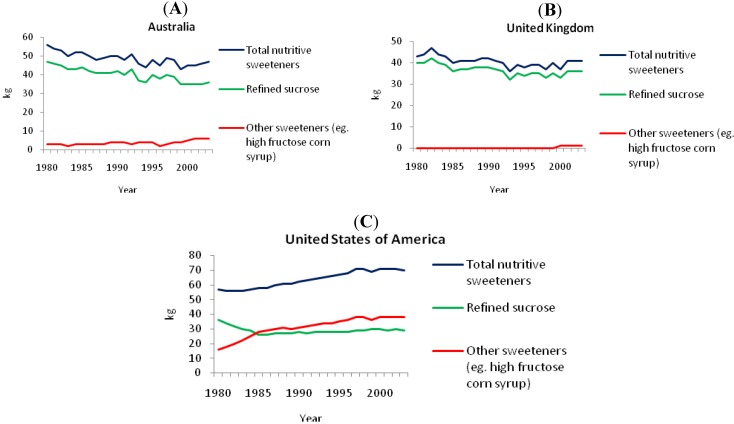
Intake of added sugars (kg/capita/year) in (**A**) Australia, (**B**) the United Kingdom and (**C**) the United States of America [25].

Figure 2 shows the refined and added sugars consumption (kg/capita/year) for Australia, the UK and the USA from 1980 to 2003 [25]. Over the period 1980-2003 in Australia, consumption of total nutritive sweeteners fell 16% (9 kg, or 25 g per day), refined sucrose consumption dropped 23% (11 kg) and consumption of other sweeteners (glucose, dextrose, fructose, lactose, isoglucose, maltose, maple sugar or similar) increased from a small baseline to 3 kg per capita (8 g per day). Over this same period in the UK, consumption of total nutritive sweeteners fell 5% (2 kg, or 6 g per day), refined sucrose consumption dropped 10% (4 kg) and consumption of other sweeteners increased to 1 kg per capita from zero consumption in 1980. In the USA, from 1980 to 2003, consumption of total nutritive sweeteners increased 23% (13 kg = 37 g per day), refined sucrose consumption dropped 20% (7 kg) while consumption of other sweeteners (primarily high fructose corn syrups in the USA) increased 138% (22 kg). In all three countries, the consumption of refined sucrose showed a downward trend [25]. 

**Figure 3:**
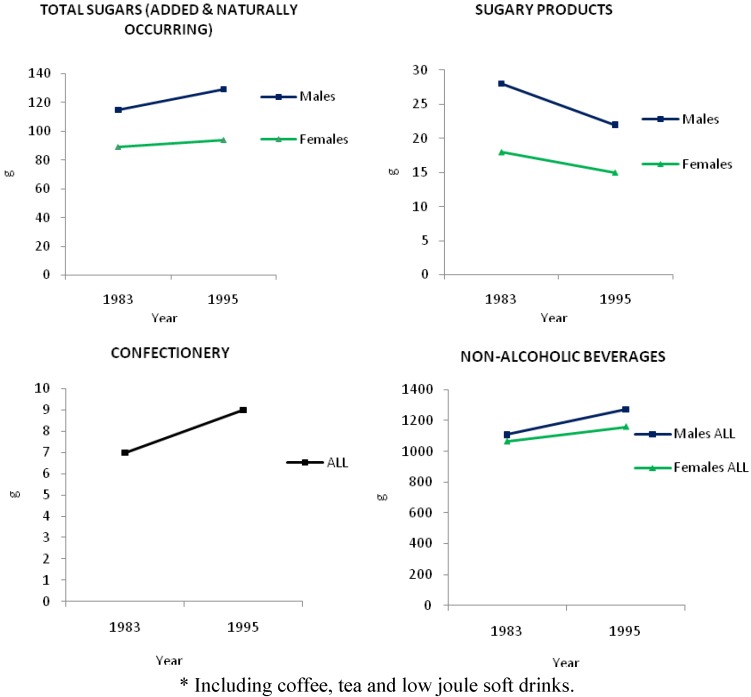
24 h mean intake (g) of total sugars, sugary products, confectionery and non-alcoholic beverages * by Australian adults (25-64 years) in 1983 and 1995 [19].

Figure 3 shows an historical comparison of the 24 h sugars intakes from various sources for Australian adults in 1983 and 1995 (most recent data available) [19]. For men, intake of total sugars (added and naturally occurring sugars in fruit, fruit juices, *etc*.) increased by 12% to 129 g from 1983 to 1995. For women, intake of total sugars increased by 6% to 94 g over this period. With regard to confectionery mean 24 h intake increased from 7 g to 9 g for both men and women from 1983 to 1995. Although the intake of confectionery showed an upward trend, absolute intake was small (<10 g) in comparison with the other sources. Intake of sugary products (e.g., cakes, cookies) decreased from 28 g to 22 g and 18 g to 15 g for both men and women respectively. For men, intake of all non-alcoholic beverages (including fruit and vegetable juices, cordials, tea and coffee, mineral waters, electrolyte drinks, sugar and non-nutritively sweetened soft drinks) increased by 15% to 1274 g from 1983 to 1995. For women, intake of all non-alcoholic beverages (including non-nutritively sweetened varieties) increased by 9% to 1159 g over the same time period. 

**Figure 4:**
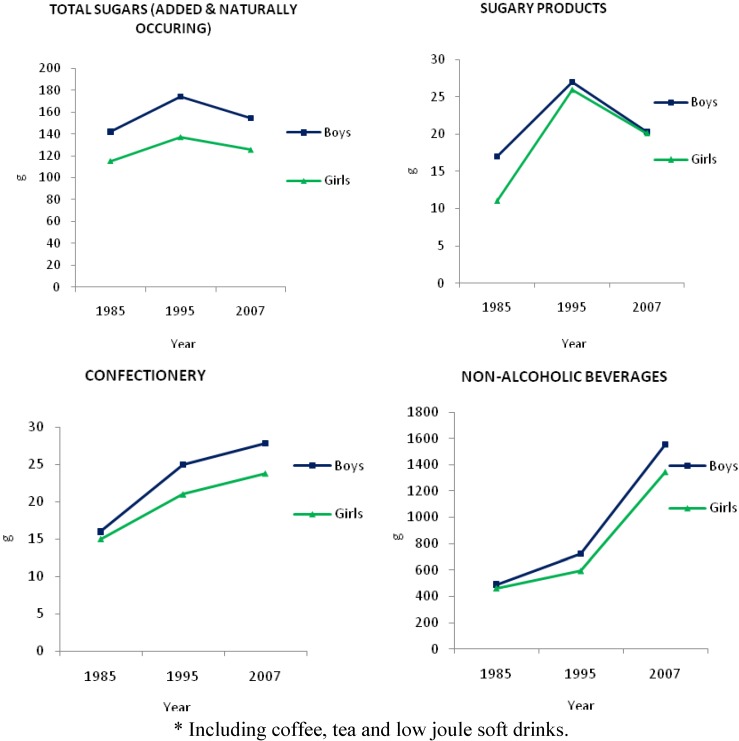
24 h mean intake (g) of total sugars, sugary products, confectionery and non-alcoholic beverages * by Australian children in 1985, 1995 and 2007 [5,19]. Note: the age categories used for comparison where 10-15 year old children in years 1985 and 1995, the 2007 figure is an average between intakes of 9-13 year and 14-16 year categories.

Figure 4 shows a comparison of the 24 h mean sugars intakes for children. For boys, intake of total sugars (added and naturally occurring) increased from 142 g in 1985 to 174 g in 1995 [19], but then declined to 154 g in 2007 [5]. Girls showed a similar pattern of intake of total sugars, with an increase from 115 g in 1985 to 137 g in 1995, then a fall to 125 g in 2007. In boys, intake of confectionery showed an increasing intake (16 g to 25 g to 28 g) for the years 1985, 1995 and 2007 respectively. Girls showed a similar increasing intake (15 g to 21 g to 24 g) across 1985, 1995 and 2007. 

In boys, the intake of sugary products increased from 17 g in 1985 to 27 g in 1995 but decreased to 20 g in 2007. Girls’ intake increased from 11 g in 1985 to 26 g in 1995 but decreased to 20 g in 2007. For boys, intake of non-alcoholic beverages (including fruit and vegetable juices, cordials, tea and coffee, mineral waters, electrolyte drinks, sugar and non-nutritively sweetened soft drinks) increased from 490 g to 724 g to 1555 g across 1985, 1995 and 2007. For girls, intake of non-alcoholic beverages (non-nutritively sweetened varieties) increased from 459 g to 592 g to 1342 g across 1985, 1995 and 2007. 

### 3.3. Nutritively Sweetened Beverage Consumption from Sales Data

**Figure 5:**
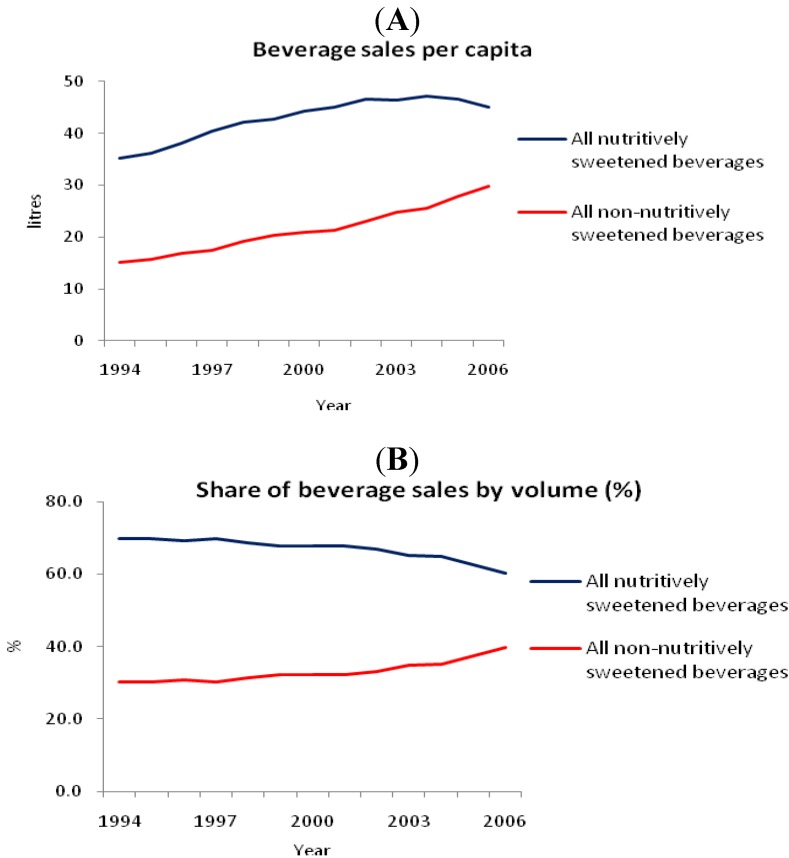
Time trends in sales of nutritively sweetened beverages and non-nutritively sweetened beverages in grocery stores, expressed as (**A**) per capita volume sold in liters and as (**B**) a percentage of total volume sold [15,28,29,30].

Figure 5 shows the time trend in sales of nutritively sweetened and non-nutritively sweetened beverages in Australian grocery stores, expressed as total volume per capita [15,28,29,30]. Nutritively sweetened beverages made up the largest share of total water-based beverage sales, but during the period 2002-2006 there was a downward trend, with an absolute reduction in sales of nutritively sweetened beverages by 64 million liters. For nutritively sweetened beverages, sales were 96 mL/day/person in 1994, increasing to 129 mL/day/person in 2004, then decreasing to 125 mL/day/person in 2006. The sales of non-nutritively sweetened (diet/low-joule) beverages increased by 34% from 1997 to 2006 [30]. Per capita, non-nutritively sweetened beverages sales doubled from 41 mL/day/person in 1994 to 82 mL/day/person in 2006 [15,28]. 

**Figure 6:**
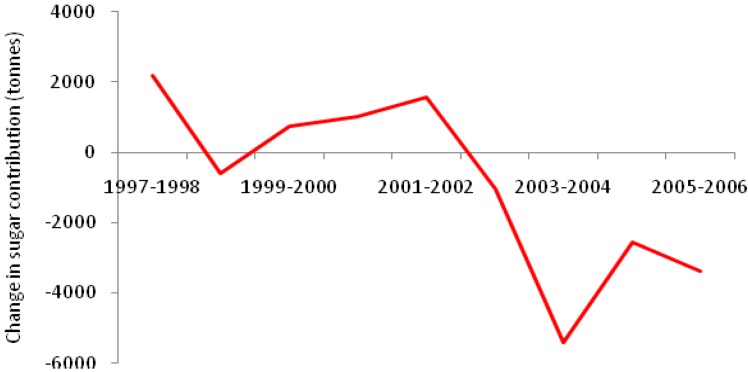
Annual change in contribution of nutritively-sweetened carbonated soft drinks to total added sugar in the Australian food supply [30].

Figure 6 shows the annual change in the contribution of sugar from nutritively sweetened carbonated soft drinks (sugar-sweetened soft drinks) to the Australian food supply [30]. Levy and Tapsell [30] reported a concurrent increase in sugar from other nutritively sweetened beverages (e.g., sports drinks, flavored waters and iced teas). However, the increase in sugar contribution to the food supply from these beverages did not contribute enough volume to match the decline in nutritively sweetened carbonated soft drinks. Overall, there was a decrease in sugar contribution from nutritively sweetened carbonated soft drinks to the Australian food supply, amounting to 12,402 tons (~600 g per person per year, Figure 6) from 2002 to 2006. 

### 3.4. Consumption of Soft Drinks, Flavored Waters, Electrolyte Drinks and Fruit Juice by Children

**Figure 7:**
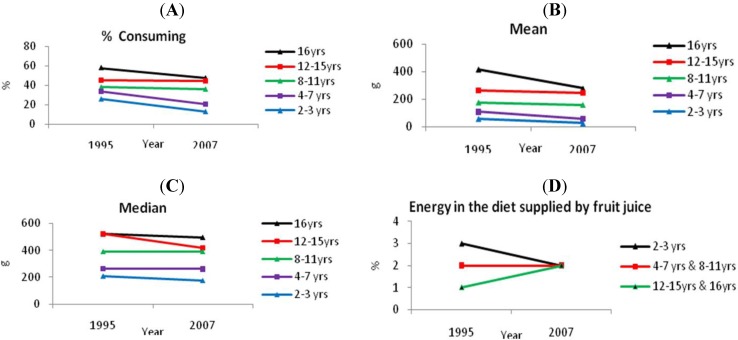
(**A**) Percent consuming, (**B**) mean intakes and (**C**) median intakes of soft drinks, flavored waters and electrolyte drinks by children in 1995 and 2007, and (**D**) percentage of energy supplied by fruit juice in the diets of children [5,26].

Overall, the percentage of children who consumed soft drinks, flavored waters and electrolyte drinks (both sugar and non-nutritively sweetened) declined from the 1995 NNS to the 2007 Australian National Children’s Nutrition and Physical Activity Survey [5,26] (Figure 7A). Among consumers, mean and median intakes of soft drinks, flavored waters and electrolyte drinks also decreased (Figure 7B). In the 16-18 year age group, mean intake fell by 33% to 278 g. In the 12-15 year age group, mean intake fell by 6% to 247 g in 2007. In the 8-11 year age group mean intake fell by 10%. In the 4-7 year age group mean intake fell by 45%. For the 2-3 year age group, mean intake fell by 55% to 26 g in 2007.

Similarly, median intake (in grams) of soft drinks, flavored waters and electrolyte drinks decreased across all age groups apart from the 8-11 year group which remained static at 391 g/day. 

Fruit juice consumption is also of interest because, like soft drinks, they represent sugars in an acidic solution. The percentage of energy supplied by fruit juice was small (of the order of 1-3% depending on age group) and changes between 1995 and 2007 were small.

## 4. Discussion

This analysis of apparent consumption, national dietary surveys and food industry data indicates a consistent and substantial decline in total refined or added sugar consumption by Australians over the past 30 years. In this respect, Australia may be unique, although FAO statistics suggest a modest reduction in refined sugar intake has also occurred in the UK. These trends contrast with a sizeable increase in the intake of total nutritive sweeteners in the USA, attributable to increased intake of high fructose corn syrup. Notably, Australia is a major grower and exporter of sugar cane, and the majority of nutritive sweetener use is in the form of refined sucrose [31].

Over the same timeframe, like other developed nations, Australia has experienced a 3-fold increase in the prevalence of obesity among adults and children. Hence in this ecological analysis, trends in refined sugar consumption are inversely related to incremental weight gain in the population as a whole. These findings support the supposition that once total energy intake has been accounted for, per capita changes in energy from sweeteners do not explain changes in the incidence of obesity [32]. Studies using individual dietary intakes have also reported inverse associations between sugar intake and body weight [33,34]. In Australia, two independent analyses of the most recent National Nutrition Survey reported no significant associations between intakes of sugars and health status, including body fatness, BMI and blood pressure [35,36]. Finally, while Australia already has some of the highest rates of overweight/obesity in the world [4,5], we are unable to rule out the possibility that rates may have be higher if consumption of sugars had not decreased over the past few decades. 

Our findings suggest that Australians have taken seriously public health recommendations to decrease sugars, particularly sugar-sweetened beverages. Food industry data indicate that per capita sales of low calorie (non-nutritively sweetened) beverages doubled from 1994 to 2006 while nutritively sweetened beverages decreased by 10%. At present, one in three soft drinks sold in Australia are non-nutritive [15,28,29,30]. Indeed, Australians have willingly adopted many other public health recommendations, including universal wearing of seat belts (the “click clack, front and back” campaign) and sunscreens (the “slip, slop, slap” campaign). 

Evidence for an association between sugars consumption and weight gain from clinical trials and epidemiological studies has been inconclusive. There have been four systematic reviews that have included evidence from a large range of clinical trials, cohort studies and cross-sectional analyses, that have investigated the role of sugar sweetened beverages in the development of obesity in humans aged 1-99 years [8,37,38,39]. Of these, only one [8] supported an independent role for sugar sweetened beverages in the etiology of overweight/obesity. Similarly, there have been two systematic reviews investigating the role of added sugars in the development of obesity in men and women [40,41]. When sucrose, glucose, or starch was replaced with >100 g of fructose/day, a weight gain of 0.44 kg/week was observed in adults [40], whereas there were inconsistent associations when sugars were replaced with non-nutritive sweeteners, starch and fat [41]. Larger, well designed clinical trials are needed to further investigate this relationship.

A limitation common to all ecological studies is that relationships observed for groups do not necessarily hold for individuals. In the national population surveys, the dietary methodologies employed varied from food frequency questionnaires to 24 h recall of food intake. Recall data are only a crude estimate of actual intake, especially in children where there is high day-to-day variability. For adults, the most recent nationally representative food intake data are now 15 years old. Recall precision accuracy, low response rates, reporting and classification errors were relatively common and may have introduced confounding. Per capita consumption data are useful in determining upward or downward trends over time and for filling gaps by describing current levels of sugar intake for the entire population. Like all apparent consumption data, there are limitations in describing individual intake due to losses that occur when foods are actually prepared and consumed (e.g., plate wastage). Indeed, Baghurst and colleagues found that intake data from several population surveys indicated that the mean level of consumption of refined sugars was not as high as was estimated from apparent consumption [42]. Nonetheless, in the case of refined sugar, individuals may consciously or unconsciously underestimate intake of a substance that is considered unhealthy. Because refined sugar is a highly controlled commodity that is not grown for personal use, apparent consumption data are perhaps the most objective way to assess trends over time. Per capita food consumption statistics from FAO have compared favorably with energy and macronutrient intake estimated from population surveys [32,43]. 

Finally, data generated by the food and beverage industry for its own purposes may not be entirely reliable because there is no independent monitoring or peer review. However, industry makes financial decisions based on consumer demand and buying patterns and there is no reason to believe that it does not reflect the true state of affairs. Their data provide information on product usage that, combined with direct intake data, provide useful insights into the food environment. 

Our findings do not support the widely held belief that reducing the consumption of refined sugars, and increasing the availability and preference for low-joule beverages, will help to reverse societal trends in obesity. Most recently, the American Heart Association stated that “added sugars are an important factor in the obesity crisis” and set strict guidelines for added sugar intake [44]. Specifically the guidelines recommend that Americans should eat or drink no more than 5 teaspoons (25 g) of added sugar per day for most women and 9 teaspoons (45 g) per day for the majority of men. 

Clearly, overconsumption of energy relative to needs must be addressed to halt the obesity epidemic. However, a recent analysis of Australian children’s dietary intakes from 1995 to 2007 revealed a substantial decrease in sugar-sweetened beverage (halved as a percentage of energy) consumption over the past decade, but increased consumption in the proportion of energy from chocolate, cakes and cookies, pizza and packet chips [33]. Furthermore, the 2007 National Children’s Nutrition and Physical Activity Survey showed that sugar and sugary beverages were not predominant “extra” foods in the diets of Australian children. Therefore, the question of whether there is much to be gained by focusing public health policy on the removal of sugar and sugar-sweetened beverages remains. The concern is that potentially more important determinants of obesity are being overlooked by the current emphasis on sugars and soft drinks.

Questioning the priority of public health messages is relevant. It is possible that less emphasis has been given to disseminating the message of lowering total energy intake, while avoidance of particular nutrients, such as sugars, has been the primary focus. In practice, many individuals over-consume “fast” foods along with a diet drink. Interestingly, research by WHO found that the Australian energy supply has increased almost exclusively as a result of an increase in intake of fat [32]. Likewise, strategies aimed at reduction of added sugars consumption alone will not automatically improve overall dietary quality [45]. Indeed, lower relative fat consumption was obvious in the high added sugars consumers (the sugar fat seesaw), which would suggest that a reduction in added sugars might lead to increased fat consumption. Logic tells us that an inappropriately high intake of any energy source (alcohol, fat, protein, starch or sugar) will result in weight gain. 

Indeed, a literal interpretation of our findings would suggest that reductions in sugar intake may have contributed to the rise in obesity. Lowering the sugar content of foods may be counterproductive for weight management if there is replacement of sugars with refined or high glycemic index starches, saturated fats or alcohol. 

## 5. Conclusions

The present analysis indicates the existence of an Australian Paradox, i.e., an inverse relationship between secular trends in the prevalence of obesity prevalence (increasing by ~300%) and the consumption of refined sugar over the same time frame (declining by ~20%). The findings challenge the implicit assumption that taxes and other measures to reduce intake of soft drinks will be an effective strategy in global efforts to reduce obesity. 

## Supplementary Files

Correction Correction published at 9 August 2011: http://www.mdpi.com/2072-6643/3/8/734/ (PDF, 27 KB)

Supplementary File 2 Editorial (PDF, 82 KB)

Supplementary File 3 Correspondence (PDF, 163 KB)
