# Barclay, A.W. and Brand-Miller, J. The Australian Paradox: A Substantial Decline in Sugars Intake over the Same Timeframe that Overweight and Obesity Have Increased. *Nutrients* 2011, *3*, 491-504

**DOI:** 10.3390/nu3080734

**Published:** 2011-08-09

**Authors:** Alan W. Barclay, Jennie Brand-Miller

**Affiliations:** 1 Australian Diabetes Council, 26 Arundel Street, Glebe, NSW 2037, Australia; Email: awbarclay@optusnet.com.au; 2 School of Molecular Bioscience and Boden Institute of Obesity, Nutrition and Exercise, University of Sydney, NSW 2006, Australia

We have found some errors in our paper published in Nutrients [1]. We found that reference 18 in section 2.2 should be removed, the reference 18 in section 3.1 should be replaced with reference 19 and the reference 33 should be corrected as follows. 

33. Rangan, A.M.; Kwan, J.; Flood, V.M.; Louie, J.C.Y.; Gill, T.P. Changes in “extra” food intake among Australian children between 1995 and 2007. *Obes. Res. Clin. Pract.***2011**, *5*, e55-e63.

We would like to apologize for any inconvenience caused to our readers.
