# Barclay, A.W. and Brand-Miller, J. The Australian Paradox: A Substantial Decline in Sugars Intake over the Same Timeframe that Overweight and Obesity Have Increased. *Nutrients* 2011, *3*, 491-504

**DOI:** 10.3390/nu6020663

**Published:** 2014-02-12

**Authors:** Alan W. Barclay, Jennie Brand-Miller

**Affiliations:** 1 Australian Diabetes Council, 26 Arundel Street, Glebe, NSW 2037, Australia; E-Mail: awbarclay@optusnet.com.au; 2 School of Molecular Bioscience and Boden Institute of Obesity, Nutrition and Exercise, University of Sydney, NSW 2006, Australia

We have found three inadvertent errors in our paper published in Nutrients [1].

- On page 498, text line 8, the words in brackets “~600 g per person *per year*, Figure 6” should be amended to “~600 g per person, Figure 6”.
- On page 500, text line 17, some words were missing. The amended sentence reads “Food industry data indicate that per capita sales of low calorie (non-nutritively sweetened) beverages doubled from 1994 to 2006 while *market share of* nutritively sweetened beverages decreased by 10% *points*.”
- On page 502, line 2, the words “increasing *by 300%*” should be amended to “increasing *3-fold*”.

These changes have no material impact on the conclusions of our paper. We apologize to our readers. 
