# Supplementary material for: The Australian Paradox: A Substantial Decline in Sugars Intake over the Same Timeframe that Overweight and Obesity Have Increased
Source: Nutrients. 2011 Apr 20;3(4):491–504. doi: 10.3390/nu3040491 (PMC3257688; doi:10.3390/nu3040491)
Supplement: Correction — published at 9 August 2011: http://www.mdpi.com/2072-6643/3/8/734/ (PDF, 27 KB) [file nutrients-03-00491-s001.pdf]

Nutrients **2011**, *3*, 734; doi:10.3390/nu3080734

OPEN ACCESS

nutrients

ISSN 2072-6643

www.mdpi.com/journal/nutrients

Correction

Barclay, A.W. and Brand-Miller, J. The Australian Paradox: A Substantial Decline in Sugars Intake over the Same Timeframe that Overweight and Obesity Have Increased. *Nutrients* 2011, *3*, 491-504

Alan W. Barclay [1] and Jennie Brand-Miller [2,*]

1 Australian Diabetes Council, 26 Arundel Street, Glebe, NSW 2037, Australia; E-Mail: awbarclay@optusnet.com.au

2 School of Molecular Bioscience and Boden Institute of Obesity, Nutrition and Exercise, University of Sydney, NSW 2006, Australia

* Author to whom correspondence should be addressed; E-Mail: j.brandmiller@mmb.usyd.edu.au; Tel.: +61-2-9351-3759; Fax: +61-2-9351-6022.

Received: 29 July 2011 / Published: 9 August 2011

We have found some errors in our paper published in Nutrients [1]. We found that reference 18 in section 2.2 should be removed, the reference 18 in section 3.1 should be replaced with reference 19 and the reference 33 should be corrected as follows.

33. Rangan, A.M.; Kwan, J.; Flood, V.M.; Louie, J.C.Y.; Gill, T.P. Changes in “extra” food intake among Australian children between 1995 and 2007. *Obes. Res. Clin. Pract.* **2011**, *5*, e55–e63.

We would like to apologize for any inconvenience caused to our readers.

References

1. Barclay, A.W.; Brand-Miller, J. The Australian Paradox: A Substantial Decline in Sugars Intake over the Same Timeframe that Overweight and Obesity Have Increased. *Nutrients* **2011**, *3*, 491–504.

© 2011 by the authors; licensee MDPI, Basel, Switzerland. This article is an open access article distributed under the terms and conditions of the Creative Commons Attribution license (http://creativecommons.org/licenses/by/3.0/).
