# Supplementary material for: The Australian Paradox: A Substantial Decline in Sugars Intake over the Same Timeframe that Overweight and Obesity Have Increased
Source: Nutrients. 2011 Apr 20;3(4):491–504. doi: 10.3390/nu3040491 (PMC3257688; doi:10.3390/nu3040491)
Supplement: Supplementary File 3 — Correspondence (PDF, 163 KB) [file nutrients-03-00491-s003.pdf]

Correspondence

The Australian Paradox Revisited

Jennie Brand-Miller [1,]* and Alan W. Barclay [2]

[1] School of Molecular Bioscience and Boden Institute of Obesity, Nutrition and Exercise, University of Sydney, Sydney, NSW 2006, Australia

[2] Australian Diabetes Council, 26 Arundel Street, Glebe, NSW 2037, Australia; E-Mail: alan@australiandiabetescouncil.com

* Author to whom correspondence should be addressed; E-Mail: jennie.brandmiller@sydney.edu.au; Tel.: +61-2-9351-3759; Fax: +61-2-9351-6022.

Received: 25 March 2012 / Published: 30 March 2012

The *Australian Paradox* reported the observation that upward changes in the prevalence of overweight and obesity in Australia run counter to changes in *refined* sugars intake [1]. Economist, Rory Robertson claims there is no Australian Paradox, just unreasonable treatment of the available data [2]. Unfortunately, there are factual errors in Mr. Robertson's essay and misinterpretation of the distinctions between total sugars *vs.* refined sugars, sugar availability *vs.* apparent consumption, sugar-sweetened and diet soft drinks, and other nutrition information. While the terminology, strengths and limitations of various nutrition data are readily understood by individuals trained in nutrition, some confusion may have been avoided if our original paper had referred to refined sugars in its title and described the terminology used.

Our peer-reviewed published analysis argued the case for a decline in refined sugar (sucrose) consumption by Australians over past decades. By several indicators, it has decreased over the same timeframe that the prevalence of overweight and obesity has risen strongly. This paradox challenges the current focus on sources of refined sugar, sucrose or fructose as primary players in the development of overweight and obesity in Australia.

The Australian Paradox relied on three independent lines of evidence: national dietary surveys, apparent consumption data from the United Nations Food and Agricultural Organisation (FAO) and beverage industry data.

National Dietary Surveys provide the most reliable estimates of food intake and therefore *total sugars*, but they do not tell us exactly what proportion is refined sugar. Total sugars intake includes the naturally-occurring sugars in fruits, vegetables, milk and dairy products as well as "added" sugars. Australian Dietary Guidelines recommend two serves of fruit a day, equivalent to ~20–30 g natural sugar, and 5 serves of vegetables, providing ~20–25 g. A glass of milk contains 12 g natural milk sugar, and 2–3 serves are recommended. A healthy diet could contain 90 g of naturally-occurring sugars.

In the last National Nutrition Survey in 1995 [3], 95% of Australian adults consumed less than 115 g of total sugars per day, a level well within international guidelines of <25% energy [4]. Although there were small increases in *total* sugars from 1983 to 1995, there were sharper declines in "sugary products" that contribute *refined* sugar to the diet. Importantly, intake of fruit and vegetables

increased [5], implying that the absolute intake of refined sugars had declined. The percentage of energy from total sugars remained either the same or decreased from 1983 to 1995, depending on the age group. Knowledge of food composition is critical to interpretation of the data.

In the 2007 national survey of Australian children [6], total sugars (naturally-occurring and added) amounted to 99, 112, 135 and 145 g per day for children aged 2–3, 4–8, 9–13 and 14–16 years, respectively. On average, they consumed ~16 g/day *less* sugar from all sources in 2007 compared to 1995 [5,6]. In 1995, they consumed 3.3% of energy as sugar-sweetened beverages; in 2007, the figure across all age groups was only 1.6% [7]. This stands in stark contrast to ~18% of energy from *added* sugars alone in American adolescents, a level that *is* a cause of concern.

The Australian Paradox also utilised statistical information provided by the website FAOStat [8]. Compiled by trained professionals and accessed by business and governments for economic analysis and policy setting, FAO data permit the analysis of time trends in apparent consumption of food commodities within and between countries. Australian data on FAOStat are supplied by the Australian Bureau of Statistics using information gathered by the Australian Bureau of Agricultural and Resource Economics (ABARE). This line of evidence indicated that while per capita refined sugars consumption has steadily declined in Australia since 1970 [8,9], the USA displays a marked increase [8].

"Apparent" consumption data are estimates of food intake based on considering a country's production, imports, exports, wastage and non-food uses of particular commodities. It is at best only a crude approximation of actual food intake but every student of nutrition learns its limitations. However, applied over time and across countries, it provides valuable information about *trends* in consumption. This was the rationale for using it as one line of evidence.

Curiously, Mr. Robertson dismisses the FAOStat data, citing ABARE data instead [10] as "the only timely official information on Australia-wide 'sugar availability'". He equates sugar availability with sugar consumption (real or apparent). He makes adjustments for population increases and concludes that "the trend over the past 22 years has been up and not down". He fails to mention the trend for the previous ~20 years (1969 to 1989) where sugar availability dropped from a peak of nearly 69 kg per person per year to a low of 30 kg per capita (Figure 1). Therefore, using only ABARE data, we can conclude that overall availability of refined sugar varied widely but shows no significant trend ($p = 0.46$) during a period when rates of obesity climbed dramatically. Sugar availability, however, is a not the same as consumption. Sugar availability takes no account of food wastage, use in animal food, beer and alcohol fermentation, or in non-food industrial use, and we cannot assume that a *steady* portion is lost in this way.

Australian Bureau of Statistics Apparent Consumption data are congruent with FAOStat data indicating sugar consumption peaked just after World War 2 (54 kg per person per year) and reached its lowest point in 1998–1999 (38 kg per person per year) [9]. However, rates of overweight/obesity nearly doubled from 37% in the 1980s [11] to 60% in 2000 [12].

Mr. Robertson also fails to note that per capita availability and consumption figures for virtually all food products (poultry, seafood, dairy, cereals, fruit and vegetables), are up in the past 22 years [9]. That is not surprising because the average Australian is now significantly older, taller and heavier than they were 30 years ago [13,14]. More food energy from protein, fat and carbohydrate is required just to maintain weight. If we eat more of everything, then the proportion of energy contributed by refined sugar will not change.

Figure 1. Changes in availability of refined sugar from sugar cane (production minus exports) in Australia per capita from 1969–1970 to 2009–2010. Data were derived from statistics published by the Australian Bureau of Agricultural and Resource Economics and Sciences, ABARE [10]. Sugar availability does not account for food wastage, use in animal food, beer and alcohol fermentation, or in non-food industrial use. From 1998–1999, the Australian Bureau of Statistics no longer derived apparent consumption statistics for any foodstuff, including sugar.

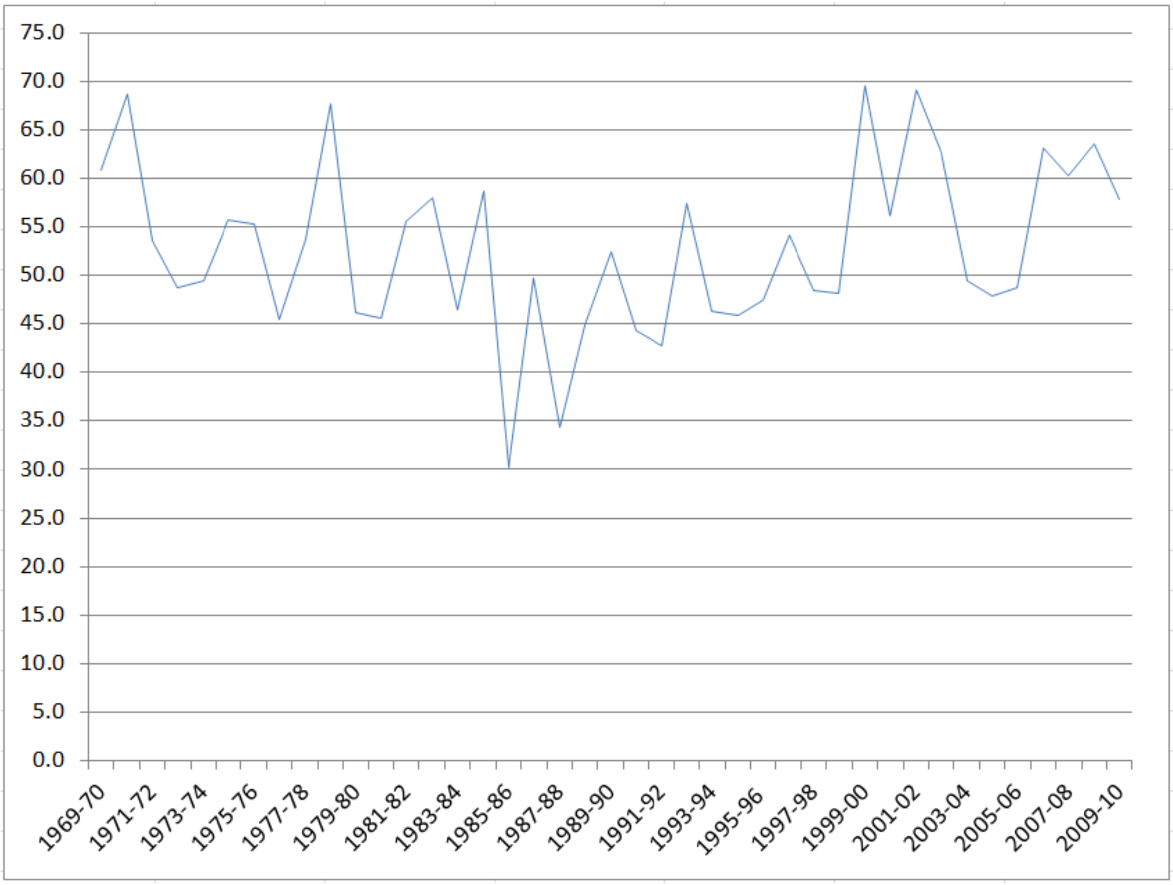

Mr. Robertson claims that total *volume* sales of soft drinks have increased but fails to distinguish soft drinks that are sugar-sweetened from those that are "diet" or low-joule flavoured waters. Diet soft drinks have increased markedly, now representing 1 in 3 soft drinks sold according to beverage industry data. Sales of "nutrient waters" with lower concentrations of sugars have also increased. In the Australian Paradox [1], we documented that sugar-sweetened sales per capita have declined *since 2003*, a period during which the prevalence of obesity continued to climb.

Considered together, these three lines of evidence are consistent with a decline in the consumption of refined sugar in Australia in recent decades.

Is Fructose a *Toxic* Carb?

Mr. Roberston expresses concerns about fructose intake in particular. The primary refined sugar used in Australia is sucrose, which yields 50% fructose by weight. Fructose also represents about ~50% of the natural sugars in fruit, vegetables and grains [15]. The large majority (95%) of

Australians consume less than 70 g of total fructose per day from all sources. Intakes greater than 100 g/day of pure *added* fructose have been suggested to cause weight gain and adverse effects on metabolism [16,17].

Two recent systematic literature reviews of all available human research on fructose [16,17] make similar conclusions "*Fructose does not seem to cause weight gain when it is substituted for other carbohydrates in diets providing similar calories. Free fructose at high doses* [*>104 g per day, or 25 teaspoons*] *that provided excess calories modestly increased body weight, an effect that may be due to the extra calories rather than the fructose.*" [17]. This evidence does not support the claim that "*sugar is toxic*".

We agree that there is no shortage of refined sugars. We agree that they can be a source of unwanted kilojoules. However, we and many other nutritionists believe refined sugar can be consumed in moderation (up to 10% of energy or ~50 g) as part of a healthy balanced diet. Often overlooked is the fact that refined starches (cornstarch, modified starches, maltodextrins) and foods high in starch such as white flour and white breads, rice, rice crackers, corn chips and crisps, can be a source of unwanted kilojoules, as well as salt and saturated fat, and devoid of essential nutrients unless fortified. There is little logic to dietary recommendations that emphasise restriction of refined sugars but ignore refined starches.

Fructose Was Not "Scarce"

From an evolutionary perspective, fruit and therefore fructose, was an important component of intelligent primate diets. Recent data show that mountain gorillas derive over 20–40% energy from fruit for 6 months of the year [18]. The anthropological literature shows that fruits that dried on the vine were available year round. In the 1980s, we analysed hundreds of traditional Aboriginal bushfoods sent to us from all over Australia, including sugarbag (bush honey) and dried bush fruits, such as the bush tomato *Solanum centrale* containing 80% sugars [19]. Like many animals, our ancestors adored honey (~70% sugars) and made sweet drinks using both honey and floral nectars [20]. Apiculture, the art of raising bees, was widely practiced even by the poor. Indeed at certain times in history, consumption of honey may well rivalled our current consumption of refined sugar [9,20]. Both evolution and anthropology therefore indicate that fructose consumption was a significant source of carbohydrate energy in human diets. Starch is the relative newcomer [21]. Nutritional analysis of a typical low GI menu generates 70 g total sugars/day, of which 35 g would be fructose.

Conclusion

Three different, independent sources of evidence indicate that Australians' intake of refined sugars intake has *not* increased. By several indicators, it has declined over the same timeframe that the prevalence of overweight and obesity has risen strongly. Australia is not alone in this regard. Over the last 10 years, other countries, including the United States, have also recorded reductions in intake of refined sugars [22,23]. This paradox challenges the view that concentrated sources of sugar, sucrose or fructose are primary players in the genesis of overweight and obesity.

Acknowledgements

JBM is a co-author of books about the glycemic index of foods (The Low GI Handbook, Hachette Livre Australia, The New Glucose Revolution, de Capo Press, New York), a Director of Glycemic Index Ltd., an international, not-for-profit GI-based food endorsement program based in Australia and supervises the University of Sydney GI testing service [24].

AB is a co-author of The Diabetes and Pre-Diabetes Handbook (Hachette Livre Australia) and a director of and consultant to Glycemic Index Ltd., an international, not-for-profit GI-based food endorsement program in program based in Australia.

References

1. Barclay, A.W.; Brand-Miller, J. The Australian Paradox: A Substantial Decline in Sugars Intake over the Same Timeframe that Overweight and Obesity Have Increased. *Nutrients* **2011**, *3*, 491–504.
2. Economist *v* nutritionists: big sugar and low-GI brigade lose. Available online: http://www.smh.com.au/business/economist-v-nutritionists-big-sugar-and-lowgi-brigade-lose-20120307-1uj6u.html (accessed on 6 March 2012).
3. Australian Bureau of Statistics; Department of Health and Aged Care Services. *National Nutrition Survey: Nutrient Intakes and Physical Measurements, Australia, 1995*; Catalogue No. 4805.0; Commonwealth of Australia: Canberra, Australia, 1998.
4. National Health and Medical Research Council. *Nutrient Reference Values for Australia and New Zealand including Recommended Dietary Intakes*; Commonwealth of Australia and New Zealand Government: Canberra, Australia, 2005.
5. Cook, T.; Rutishauser, I.H.E.; Allsopp, R. *The Bridging Study—Comparing Results from the 1983, 1985 and 1995 Australian National Nutrition Surveys*; Commonwealth Department of Health and Aged Care: Canberra, Australia, 2001.
6. Commonwealth Scientific Industrial Research Organisation (CSIRO); Preventative Health National Research Flagship; the University of South Australia. *2007 Australian National Children's Nutrition and Physical Activity Survey—Main Findings*; Commonwealth of Australia: Canberra, Australia, 2008.
7. Clifton, P.M.; Chan, L.; Moss, C.L.; Miller, M.D.; Cobiac, L. Beverage intake and obesity in Australian children. *Nutr. Metab. (Lond.)* **2011**, *8*, 87.
8. The Food and Agriculture Organisation of the United Nations. Food Consumption Quantities. 2 June 2010. Available online: http://faostat.fao.org/site/368/DesktopDefault.aspx?PageID=368#ancor (accessed on 11 August 2009).
9. Australian Bureau of Statisics. *Apparent Consumption of Food Stuffs, Australia, 1997–98 and 1998–99*; Catalouge No. 4306.0; ABS: Canberra, Australia, 1998.
10. Australian Bureau of Agricultural and Resource Economics and Sciences (ABARES) Website. Available online: http://www.daff.gov.au/abares (accessed on 23 March 2012).
11. Hodge, R.L. Risk factors in Australians: National Heart Foundation's Risk Factor Prevalence Study, 1980. *Aust. N. Z. J. Med.* **1984**, *14*, 395–399.

12. Cameron, A.J.; Zimmet, P.Z.; Dunstan, D.W.; Dalton, M.; Shaw, J.E.; Welborn, T.A.; Owen, N.; Salmon, J.; Jolley, D. Overweight and obesity in Australia: the 1999–2000 Australian Diabetes, Obesity and Lifestyle Study (AusDiab). *Med. J. Aust.* **2003**, *178*, 427–432.
13. Australian Bureau of Statistics. Animated Population Pyramid. Available online: http://www.abs.gov.au/websitedbs/d3310114.nsf/home/Population%20Pyramid%20-%20Australia (accessed on 23 March 2012).
14. Australian Bureau of Statistics. *National Health Survey: Summary of Results, 2007–2008 (Reissue)*; Catalogue No. 4364.0; ABS: Canberra, Australia, 2009.
15. Department of Health and Ageing. *NUTTAB 2006 Online*; Australian Government Publishing Service: Canberra, Australia, 2006.
16. Livesey, G.; Taylor, R. Fructose consumption and consequences for glycation, plasma triacylglycerol, and body weight: meta-analyses and meta-regression models of intervention studies. *Am. J. Clin. Nutr.* **2008**, *88*, 1419–1437.
17. Sievenpiper, J.L.; de Souza, R.J.; Mirrahimi, A.; Yu, M.E.; Carleton, A.J.; Beyene, J.; Chiavaroli, L.; Di Buono, M.; Jenkins, A.L.; Leiter, L.A.; Wolever, T.M.; Kendall, C.W.; Jenkins, D.J. Effect of Fructose on Body Weight in Controlled Feeding Trials: A Systematic Review and Meta-analysis. *Ann. Intern. Med.* **2012**, *156*, 291–304.
18. Rothman, J.M.; Raubenheimer, D.; Chapman, C.A. Nutritional geometry: gorillas prioritize non-protein energy while consuming surplus protein. *Biol. Lett.* **2011**, *7*, 847–849.
19. Brand-Miller, J.; James, K.; Maggiore, P. *Tables of Composition of Australian Aboriginal Foods*; Aboriginal and Torres Islander Studies Press: Canberra, Australia, 1993.
20. Allsop, K.A.; Miller, J.B. Honey revisited: a reappraisal of honey in pre-industrial diets. *Br. J. Nutr.* **1996**, *75*, 513–520.
21. Cordain, L.; Eaton, S.B.; Brand-Miller, J.; Lindeberg, S.; Jensen, C. An evolutionary analysis of the aetiology and pathogenesis of juvenile-onset myopia. *Acta Ophthalmol. Scand.* **2002**, *80*, 125–135.
22. Chun, O.K.; Chung, C.E.; Wang, Y.; Padgitt, A.; Song, W.O. Changes in intakes of total and added sugars and their contribution to energy intake in the U.S. *Nutrients* **2010**, *2*, 834–854.
23. Welsh, J.A.; Sharma, A.J.; Grellinger, L.; Vos, M.B. Consumption of added sugars is decreasing in the United States. *Am. J. Clin. Nutr.* **2011**, *94*, 726–734.
24. The University of Sydney GI Testing Service. Available online: http://www.glycemicindex.com (accessed on 23 March 2012).

© 2012 by the authors; licensee MDPI, Basel, Switzerland. This article is an open access article distributed under the terms and conditions of the Creative Commons Attribution license (http://creativecommons.org/licenses/by/3.0/).
